# Discovery of delayed gas production after implantation of a continuous-flow left ventricular assist device and a preliminary exploration of the mechanisms of its occurrence

**DOI:** 10.3389/fcvm.2024.1417005

**Published:** 2024-07-23

**Authors:** Pengfei Lv, Yadong Zhang, Hongbin Ma, Fenghua Wang, Ailin Chen, Zhixiang Zhang, Fan Wang, Tianwen Liu, Jiemin Zhang, Xiaocheng Liu, Zhigang Liu

**Affiliations:** ^1^ Department of Cardiac Surgery, TEDA International Cardiovascular Hospital, Chinese Academy of Medical Sciences & Peking Union Medical College, Tianjin, China; ^2^ Chinese Academy of Medical Sciences, Peking Union Medical College, Beijing, China; ^3^ Rocor Medical Technology Co., Ltd., Tianjin, China

**Keywords:** continuous-flow left ventricular assist device (CF-LVAD), gas production, pump thrombosis, computational fluid dynamics (CFD), degassing, cavitation

## Abstract

**Objective:**

To characterize the gas production phenomenon in the animal model of left ventricular assist device (LVAD), and study its mechanism.

**Methods:**

An *in vitro* bubble precipitation experiment was conducted, and the blood samples of Parma spp. animals were divided into ordinary group and oxygen-enriched group according to whether they were oxygenated or not at the time of blood collection, and a static control group was set up respectively. Blood gases were drawn and analyzed before and after the experiment. Activate the pump, and the number of air bubbles in the loop was measured by ultrasound at different rotational speeds; CFD was applied to simulate the flow field in the blood pump, and pressure, fluid velocity vector and shear force diagrams were plotted, and a thrombus model was constructed and the flow field was simulated and plotted as a cloud diagram.

**Results:**

There was a statistical difference in the number of bubbles in the inflow and outflow tubes of the blood pump (*P* values of 0.04 and 0.023, respectively), and the number of bubbles in the outflow tubes of both groups was significantly higher than the number of bubbles in the inflow tubes. The number of bubbles in the tubes of both the oxygen-enriched and normal groups was significantly higher than that in the inflow group. In both the normal and oxygen-enriched groups, more gas was produced at higher speeds than at lower speeds. Blood gas analysis showed that the reduced gas composition in the blood was mainly oxygen. Flow field simulation results: the high rotation speed group had lower central pressure and greater scalar shear. The thrombus simulation group was more prone to turbulence, sudden pressure changes, and greater shear than the normal group.

**Conclusion:**

Blood gas production is associated with higher partial pressures of blood oxygen, higher rotation speed, and intrapump thrombosis, and the mechanism of pump gas production is degassing of dissolved gases rather than cavitation of water, and the gas released is most likely to have oxygen. The degassing phenomenon is an warning factor for pump thrombosis.

## 1 Introduction

Heart failure is defined as a group of complex clinical syndromes caused by multiple structural or functional abnormalities of the heart, resulting in systolic and/or diastolic dysfunction, causing failure of the heart and various organs of the body (1). End-stage heart failure is the final stage of heart failure, known as the “cancer of heart disease”, and is the last battlefield of cardiovascular disease prevention and control (2, 3). According to the 2022 China Health Statistics Yearbook and Global Burden of Disease data, the total number of heart failure patients in the world reaches 64.3 million, and the number of heart failure patients in China is about 13 million, of which nearly one-third progresses to end-stage heart failure, with a 5-year mortality rate as high as 50% (4).

**Figure 1:**
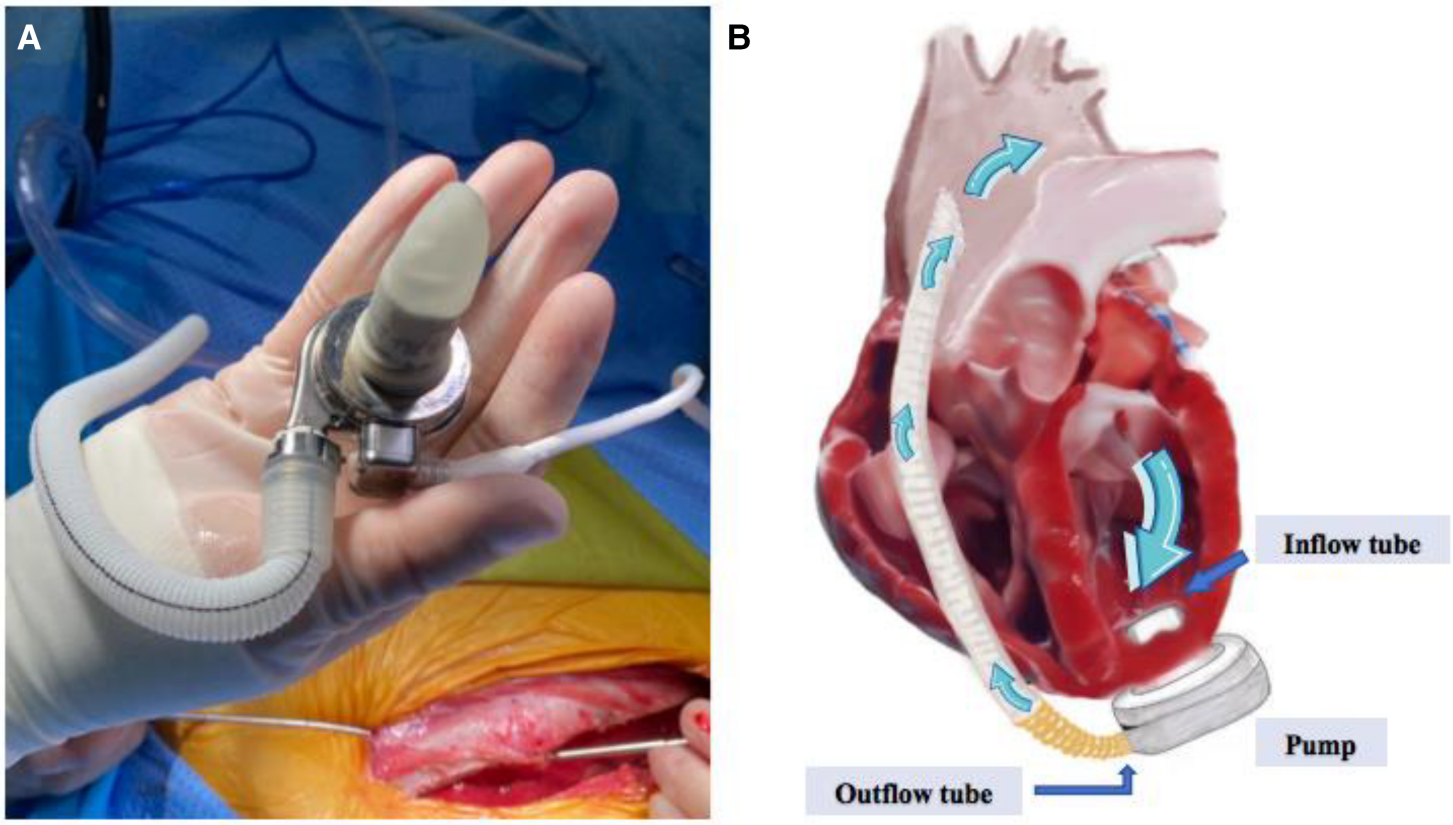
(**A**) Shows the appearance of the S prototype, (**B**) shows the working schematic of the S prototype, containing the inflow tube, the pump body, the outflow tube and the direction of blood flow.

**Figure 2:**
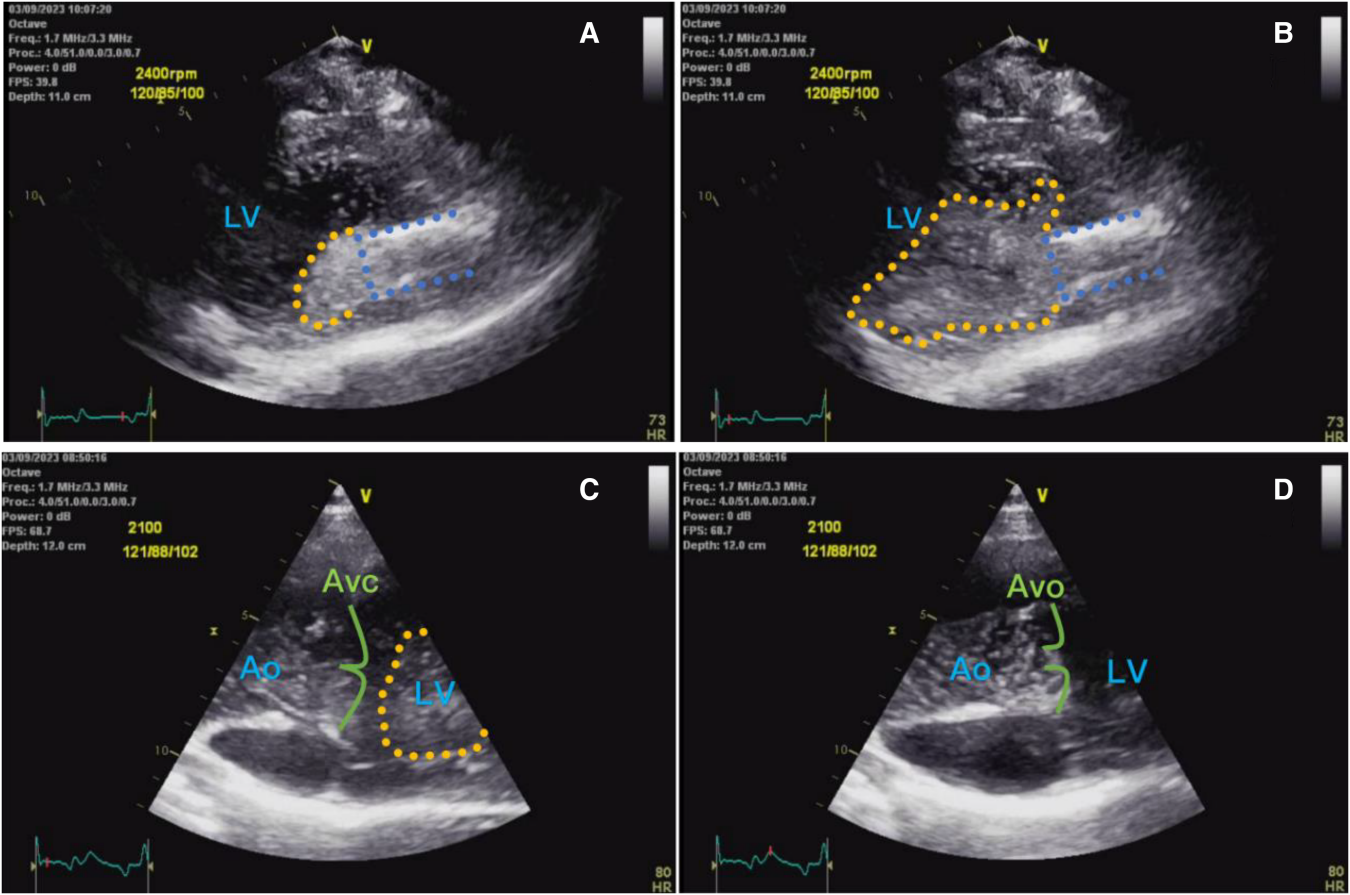
(**A**) Shows ultrasound left ventricular long-axis view of a small number of bubbles (yellow dashed area) entering the left ventricle from the pump inflow tube (blue dashed area) during early diastole; (**B**) shows a large number of bubbles entering the left ventricle from the inflow tube at end-diastole; (**C**) shows a large number of bubble clusters in the left ventricle during diastole, with a moderate number of bubbles in the ascending aorta; and (**D**) shows a large number of bubbles in the left ventricle during systole entering the ascending aorta via the aortic valve.

Artificial hearts include implantable ventricular assist device (VAD) and total artificial heart, which can be used to assist the pumping function of the heart after implantation, improve the quality of life and prolong the survival of heart failure patients. Ventricular assist devices have been widely used in the treatment of end-stage heart failure, and our center is one of the first centers in China to carry out the research and development of ventricular assist devices. In the fully magnetically levitated ventricular assist device (S prototype), as shown in Figure 1, it was found in recent animal experiments that on the day after the operation, surface ultrasound suggested that a large number of bubble clusters were continuously generated within the ventricle of an experimental sheep, entering the left ventricle from the inflow tube of the blood pump in ventricular diastole, and then entering the ascending aorta through the 1:1 open aortic valve in systole (Figure 2), the dynamic videos of ultrasound image can be seen in Supplementary Video 1 and Video 2, and the phenomenon was the first time to be detected domestically.

Due to the short application time of ventricular assist device in China, the number of surgical cases is small and lack of experience, so there is no literature report on the phenomenon of air bubbles generated in the heart after blood pump implantation and related studies. A review of the data shows that similar phenomena are rare worldwide, and most of them are reported as cases or single digits. It has been reported that gas production occurs in patients with LVAD, mostly detected during transthoracic cardiac ultrasound, and manifests itself as a variable number of microbubbles found in the aortic sinus, ascending aorta, or left ventricular heart chambers. This is a relatively new phenomenon that may be associated with pump thrombosis (5). In fact, the pump gas production phenomenon has been extensively studied in physics and is known as pump cavitation and cavitation. Cavitation refers to the local pressure within the liquid is reduced to below the saturation vapor pressure of water, liquid internal or liquid-solid interface on the formation of vapor or gas bubbles, development, collapse and collapse process. Cavitation refers to the collapse of the bubble to form micro-excursions and micro-jets, attacking the wall to form the damage process (6). However, can the LVAD axial impeller really generate such low pressure and thus cause blood cavitation? Girod et al.'s study gave a brand new idea (7). His study showed that the same bubbles are generated, either by cavitation or degassing, and the conditions under which these two mechanisms occur, as well as the sizes of the bubbles and the types of gases produced, are different. Girod judged that in the Girod determined that the bubbles found in the ascending aorta and distal carotid arteries of patients with mechanical valves were consistent with degassing and not cavitation.

Computational fluid dynamics (CFD) technique can provide insight into the hemodynamic state of the blood pump at any level, and quantitative analysis of hemodynamic patterns can visualize the possible conditions of blood gas production, such as pressure variations and shear forces; however, CFD modeling does not fully present the true hemodynamic state *in vivo*, especially when applied to the blood pump model, where the flow field is strongly affected by ventricular systolic and diastolic states. However, this limitation cannot hide the advantages of CFD, and the collection of hemodynamic parameters at different rotational speeds can help us to judge its influence on the effect of gas production. By artificially setting a thrombus module on the rotor surface, the effect of thrombus formation on the interfacial flow field can be simulated as a means of determining the effect of thrombus formation on blood pump gas production (8).

Although it is not the first time that intracardiac air bubbles have been found in ventricular assist patients, there is still a relative gap in the study of their formation mechanism, with some scholars identifying this phenomenon as cavitation, but the conditions under which it occurs have not yet been clearly elucidated. Considering that this new phenomenon may hide an unknown clinical value behind it, and that some studies have linked gas production to intrapump thrombosis as well as stroke and coronary atherosclerosis (9, 10), it should be given due attention. In order to verify the origin of intracardiac gas bubbles after implantation of ventricular assist devices, and to explore the conditions of gas production associated with blood pumps in real blood environments, the types of gases that may be present within the bubbles, and the mechanisms by which they occur, our team carried out *in vitro* validation experiments and CFD analyses.

## 2 Methods

### 2.1 Blood pump style and basic performance

Full magnetic levitation centrifugal blood pump S prototype, head 100 mmHg, flow rate 1–10 L/min. In addition to the prototype pump body, the device implantation accessories include ventricular suture ring, artificial blood vessels with a diameter of 1 cm and skeletonized protective stent, apical perforator, percutaneous wire traction device, etc. The device is designed to be used in a variety of applications. The external kit includes a controller and 2 inter-swappable high-capacity batteries. The controller data can be transmitted to the background monitoring system via Bluetooth to provide real-time feedback on the working conditions of the blood pump, including heart rate, pump speed, flow rate, power and voltage.

### 2.2 Animal blood collection and grouping

One 45 kg female Parma pig was selected, fasted and watered for 12 h before blood collection, and then anesthetized and connected to the blood collection tape via femoral artery puncture cannula for blood collection. Firstly, 600 ml of blood was collected from the pig in the state of breathing air (oxygen concentration of 21%), of which 500 ml was for the normal group and 100 ml was for the normal control group, and then 600 ml of blood was collected from the arteries again after the administration of nasal cannula oxygen for 5 min (the flow rate of the oxygen was 5 L/min, and the concentration of the oxygen was 41%), of which 500 ml was for the oxygen-enriched group, and 100 ml was for the oxygen-enriched control group. This study was carried out in the Animal Experiment Center of TEDA International Cardiovascular Disease Hospital [SYXK (Jin) 2020-0004]. It was reviewed by the Experimental Animal Ethics Committee of TEDA International Cardiovascular Disease Hospital (Ethics No. TICH-JY-20230328-1). The Regulations on the Administration of Laboratory Animals (Amended Version, March 1, 2017) issued by the State Council of the People's Republic of China and the guidelines for the care and use of laboratory animals were strictly followed.

### 2.3 Assembling the experimental pipeline and exhausting

**Figure 3:**
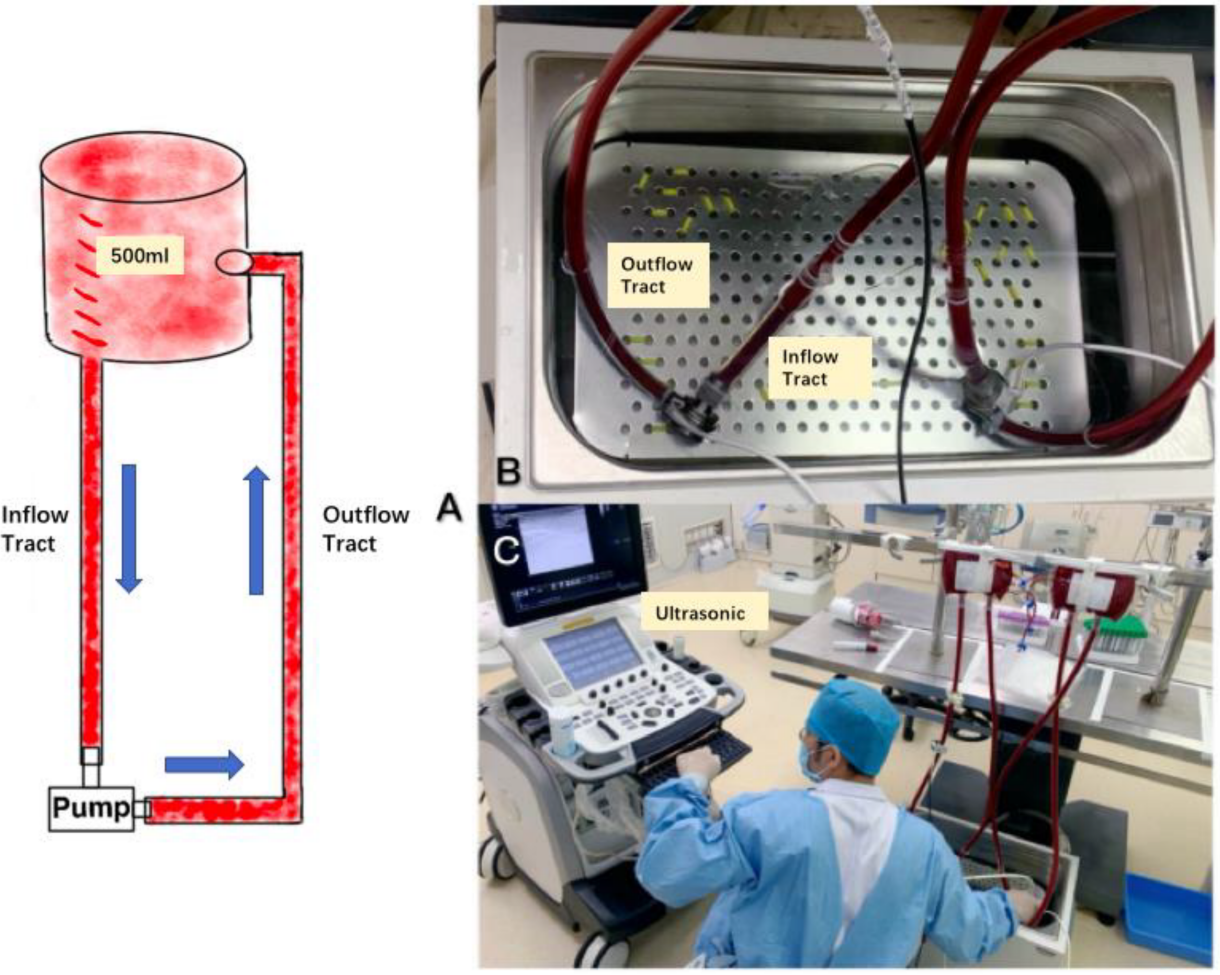
(**A**) Shows the *in vitro* experimental line, (**B**) shows the pump placed in a 37°C water bath to maintain a constant temperature, and (**C**) shows the number of bubbles generated within the diagnostic line through ultrasound.

The blood collection bag and the blood pump were connected through the silicone tube containing heparin anticoagulant coating to form a closed loop (Figure 3A), and the gas in the loop was exhausted by tapping + suction method to reach the standard of no bubbles visible to the naked eye and ultrasound. Both blood pumps were placed in a thermostatic water bath (Figure 3B) and maintained at 37°C to simulate the *in vivo* ambient temperature.

### 2.4 Ultrasonic diagnostic instrument to record the number of bubbles

Before the experiment, blood gases were extracted and analyzed to compare the partial pressures of blood oxygen of the two groups of samples. 2 min after starting the pump at 2,000 rpm, 3 consecutive sets of recordings were made using the ultrasound probe on the inflow and outflow tubes of the two experimental groups and left the graphs, and then adjusted the rotational speed to 2,500 rpm and waited for 2 min, and 3 consecutive sets of recordings were made using the ultrasound probe on the inflow and outflow tubes of the two experimental groups and left the graphs, and finally, the rotational speed was Finally, the rotational speed was adjusted to 3,000 rpm, and the experimental results were recorded in the same way as in the previous two experiments. After all the ultrasound images were retained, the air bubbles in the ultrasound images were counted according to each standard section area (Figure 3C).

### 2.5 CFD

**Figure 4:**
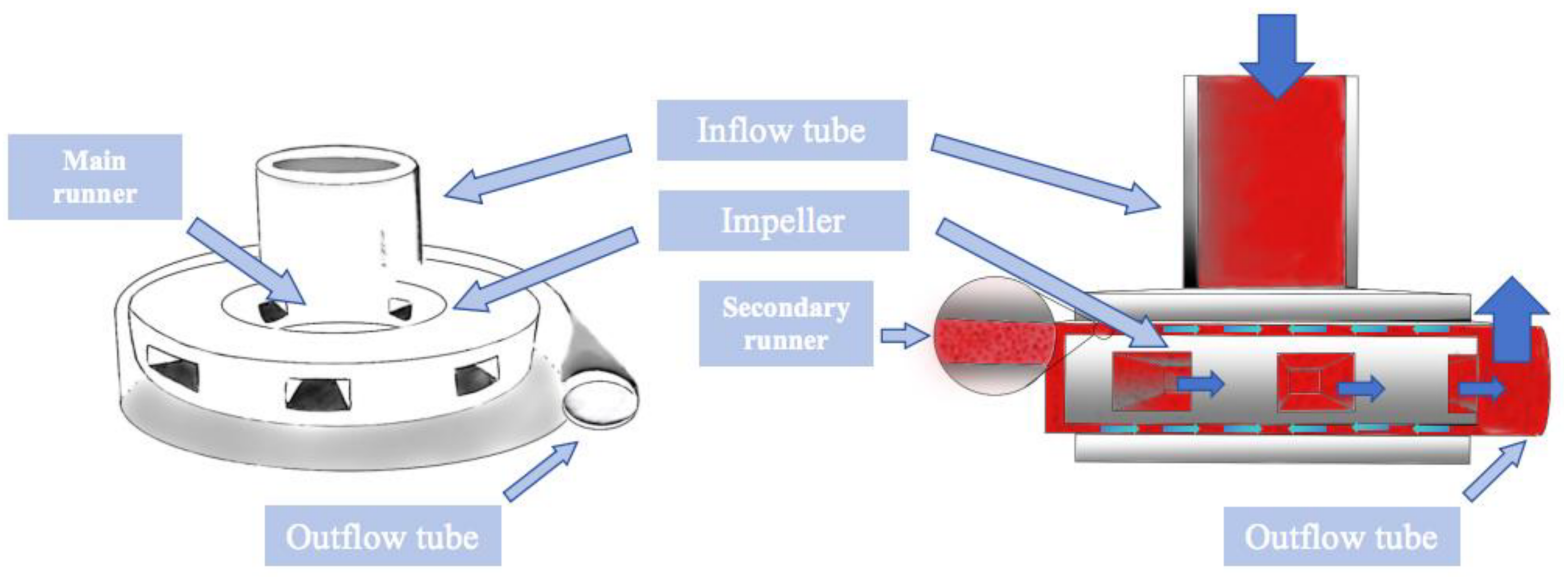
Schematic diagram of the internal composition of the blood pump and the route of blood flow operation.

Figure 4 is a schematic diagram of the blood flow through the main components of the experimental full magnetic levitation centrifugal blood pump, the blood flows in from the inflow pipe, centrifuged by the five main flow channels of the rotor, and then flows out through the outflow pipe, part of the blood centrifuged to the wall of the pump will be centrifuged to the upper and lower surfaces of the rotor and the worm case along the gap between the rotor and worm shell to flow back to the center of the rotor and participate in the next circulation, the gap for the blood to carry on the second circulation is called the secondary flow channel. The rotor is not in contact with the top, bottom, right and left directions in the worm shell, and is suspended, and the gap between the top and bottom surfaces and the worm shell is 200 um. The impeller has 5 blades, which support the rotor, and adopts the backward curved runner design, which generates the speed and pressure of the blood flow through high-speed rotation, and at the same time, guides the blood flow to enter into the direction of flow of the rotor. The pump is designed to rotate at a speed of 1,500–3,500 rpm to provide a static pressure of blood greater than 100 mmHg (1 mmHg = 133.322 Pa) at a blood flow rate of 5 L/min.

ANSYS CFX version 2022 software is applied for mesh generation, computational solution and flow field post-processing. The computational mesh of the blood pump is based on a multi-block mesh structure with a total of about 10 million meshes. Based on CFD technology, the Menter's Shear Stress Transport (SST)-k-ω type was used as the turbulence model for this solved equation (11), and according to Fraser et al.'s results (12), it was assumed that the blood was an incompressible Newtonian liquid with a density of 1,050 kg/m³, and the dynamic viscosity defined as 3.5 mpa.s. An uncoupled implicit algorithm with second-order double-precision is used to solve the following governing equations:

① Continuity equation:
  $$\frac{\partial U_{i}}{\partial X_{i}}\;=\;0$$
② Momentum equation:
  $$\rho \frac{\partial U_{j}}{\partial t}\;+\;U_{i}\rho \frac{\partial U_{j}}{\partial X_{i}}\;=\;-\;\frac{\partial P}{\partial X_{j}}\;+\;\mu \frac{\partial^{2}U_{j}}{\partial X_{i}^{2}}\;+\rho f_{j}$$

where: *i*, *j* = 1, 2, 3; $\rho \frac{\partial U_{j}}{\partial t}$ is the nonstationary term, $U_{i}\rho \frac{\partial U_{j}}{\partial X_{i}}\;$ is the convection term, $\mu \frac{\partial^{2}U_{j}}{\partial X_{i}^{2}}$ is the diffusion term, and $\rho f_{j}$ is the volume force.

Adult patients' blood pressure is 80–120 mmHg (1 mmHg≈0.133 kPa), heart rate in quiet state is about 60–80 bpm, the average is 75 bpm, output per beat is about 65 ml, cardiac output is about 5l/min, heart failure patients' left ventricular end-diastolic blood pressure is often higher than normal because of diastolic function limitation, in view of this, the static blood pressure of the inflow port of the blood pump was set at 10 mmHg, and the outlet was set at a volume flow rate of 5 L/min (13), and the rotational speeds of the smooth impeller and the impeller with a small piece of thrombus adhering to the lower surface were set at 2,000 rpm, 2,500 rpm, and 3,000 rpm, respectively, and the numerical simulation of the blood pump was performed to analyze the numerical simulation of the blood pump, and the horizontal cross-section of the middle of the impeller of the blood pump and the lower worm casing was selected, and the output of the scalar shear force cloud and the pressure and velocity vector cloud were compared.

## 3 Results

**Figure 5:**
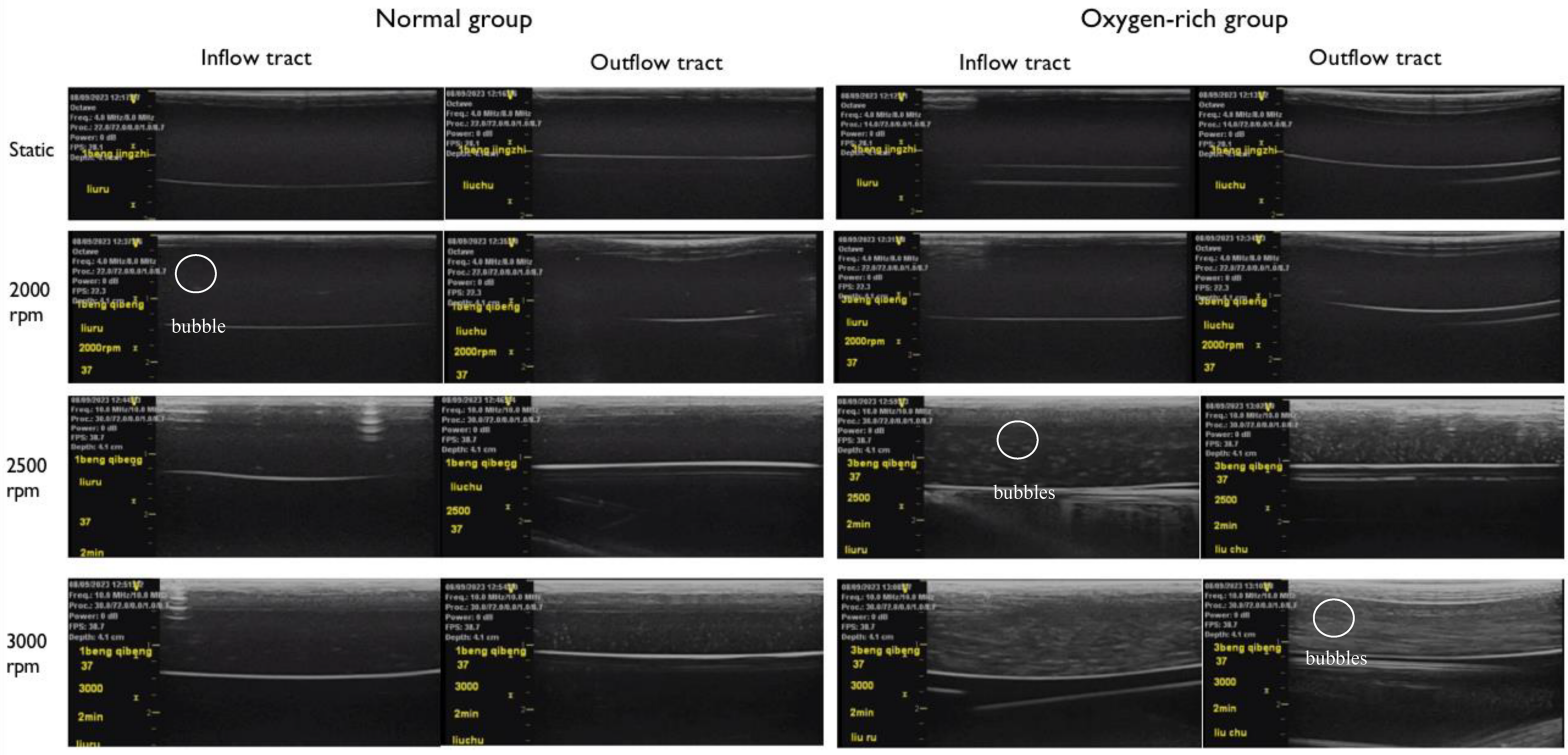
The left side is the experimental group of normal arterial blood, and the right side is the experimental group of oxygen-enriched arterial blood, and no air bubbles were found to be precipitated in the tubes in the stationary state in both groups. Comparison at the same rotational speed suggests that the oxygen-enriched group precipitates more air bubbles than the ordinary group; in the comparison within the same group, the higher rotational speed precipitates more air bubbles than the lower rotational speed.

**Figure 6:**
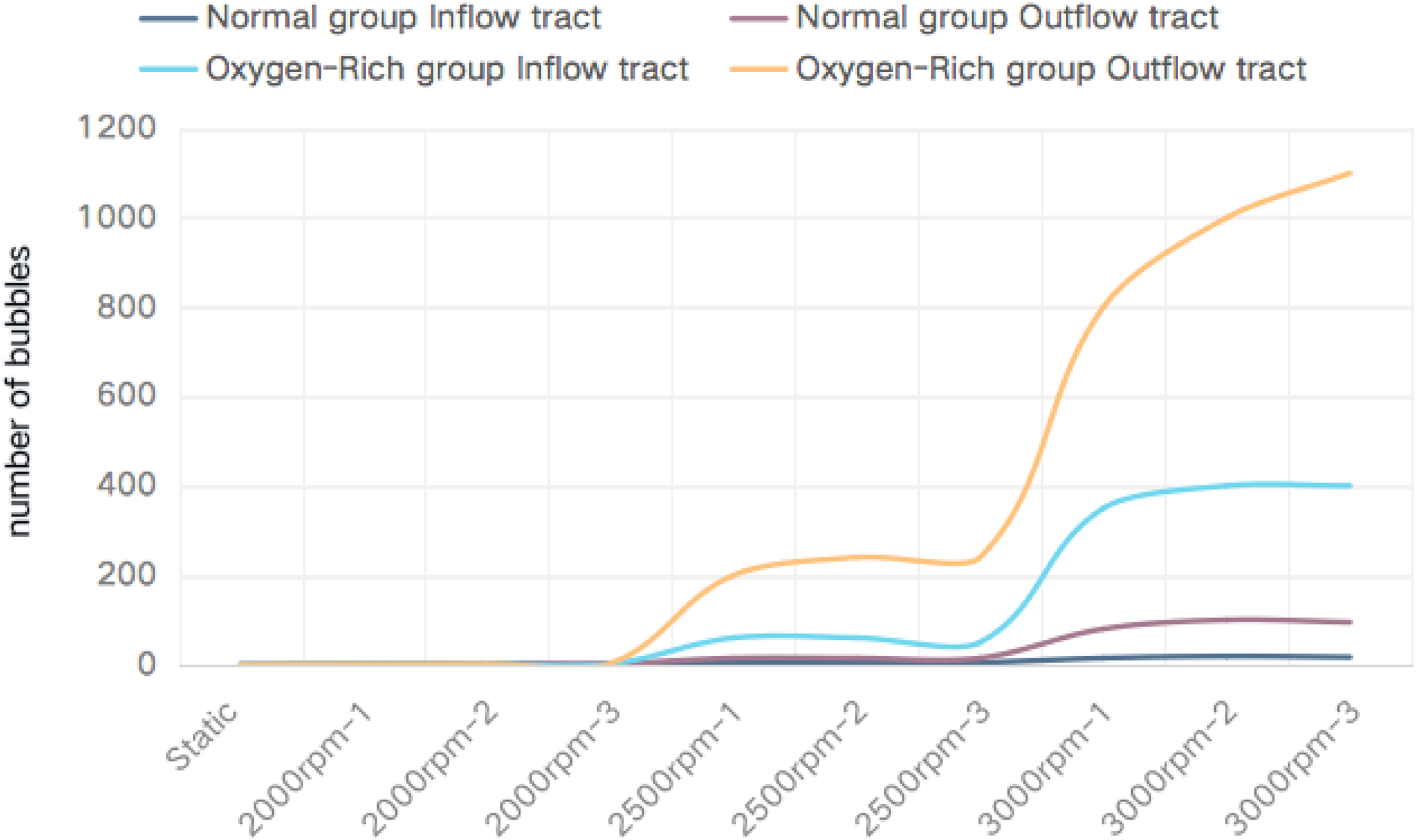
The horizontal axis is the pump speed and the vertical axis is the number of bubbles.

Blood gases of the two experimental groups and the control group were taken and analyzed before the experiment, in which the partial pressure of blood oxygen in the normal control group and the normal experimental group was 99 mmHg; and that in the oxygen-enriched control group and the oxygen-enriched experimental group was 109 mmHg. The two groups were ultrasonically inspected before the startup of the blood pump, and there were no air bubbles in all parts of the loop. The ultrasound results are shown in Figure 5, and the measurement of the number of air bubbles is shown in Table 1 and Figure 6.

**Table 1:** Bubble number metering of pre-pump(inflow tract) and post-pump(outflow tract) lines with ultrasound at three speeds, 2,000 rpm, 2,500 rpm, and 3,000 rpm in two experimental groups.

	Normal group		*P* value	Oxygen-rich group		*P* value
	Inflow tract	Outflow tract		Inflow tract	Outflow tract	
Bump-speed			0.04			0.023
Static	0	0		0	0	
2,000 rpm (2.3 L/min)			0.519			0.649
2,000 rpm-1	1	1		1	1	
2,000 rpm-2	2	2		2	2	
2,000 rpm-3	1	2		2	3	
2,500 rpm (3.6 L/min)			0.004			0.004
2,500 rpm-1	5	15		60	200	
2,500 rpm-2	4	15		60	240	
2,500 rpm-3	5	15		50	240	
3,000 rpm (5 L/min)			0.002			0.001
3,000 rpm-1	15	80		350	800	
3,000 rpm-2	19	100		400	1,000	
3,000 rpm-3	17	95		400	1,100	

Paired comparisons of the number of bubbles before and after inflow in the normal and oxygen-enriched groups were performed by paired *t*-tests, and it was found that the number of bubbles in the outflow tubes was significantly higher than that in the inflow tubes in both groups (Table 1), indicating that the intervention was effective and that the bubbles arose from within the blood pump.

By analyzing the two groups as independent samples, the results showed that the number of bubbles in the inflow and outflow tubes of the oxygen-enriched group was significantly higher than that of the normal group, indicating that the higher the oxygen content, the more pronounced the effect of gas production (Figure 6).

ANOVA analysis was conducted to examine the gas production effect at different rotational speeds in the normal group, and the results indicated that the rotational speed had an effect on the gas production effect in the normal group. Following this, a *post hoc* two-by-two comparison was performed using the Bonferroni method, and the results showed that the effect of high speed on gas production was more pronounced than that of low speed in the normal group. Similarly, in the oxygen-enriched group, the rotational speed also had a significant effect on the gas production effect, and the high rotational speed was more pronounced than the low rotational speed.

In conclusion, both in the normal group and the oxygen-enriched group, high speed is more obvious than low speed in gas production (Figure 6).

**Figure 7:**
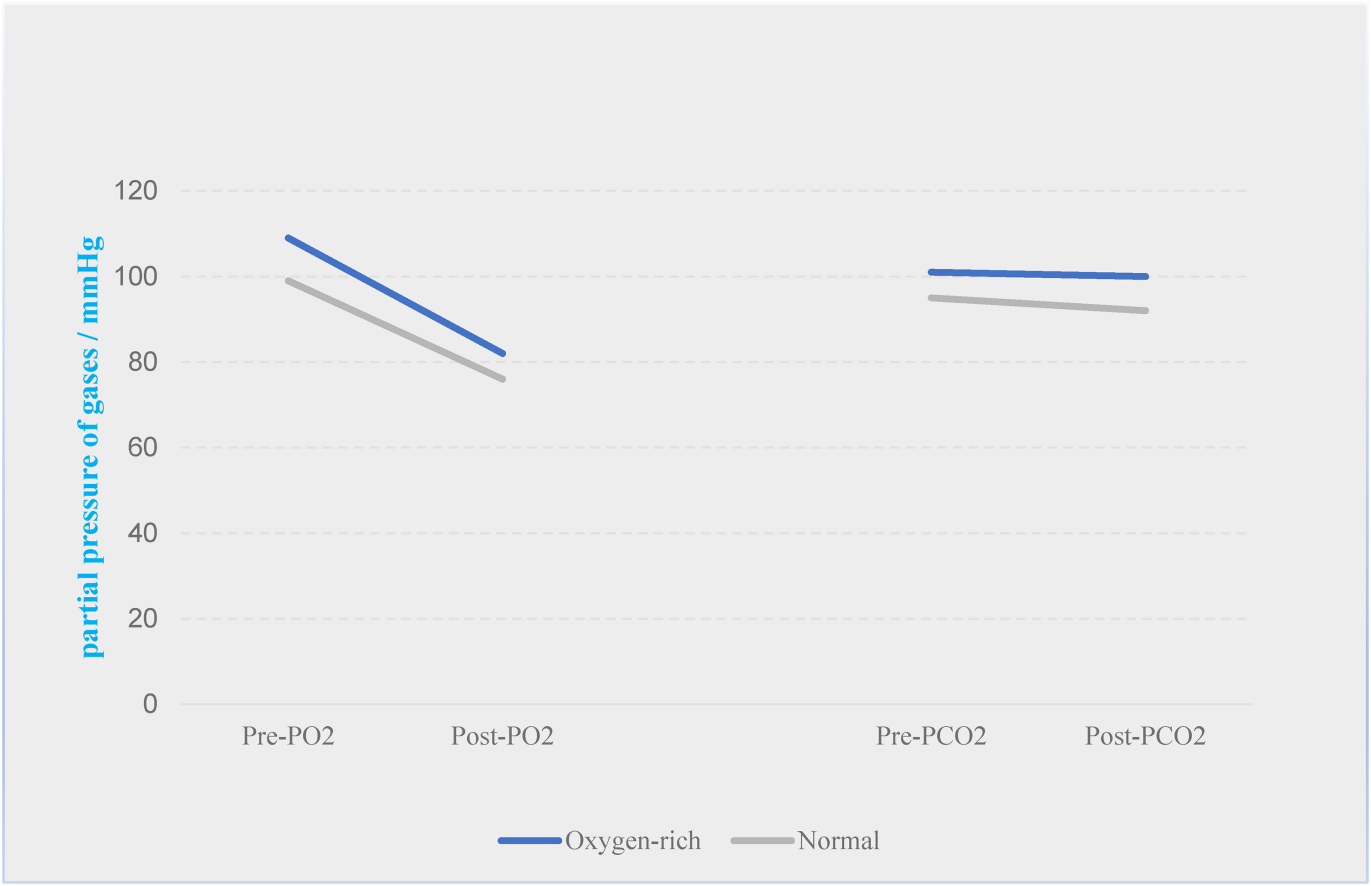
Changes in the content of the two gases in the two groups before and after the experiment.

In order to investigate the main components of the precipitated gas bubbles, the partial pressure of oxygen (PO2) and partial pressure of carbon dioxide (PCO2) in the preoperative blood gases were compared with the results of the blood gases circulated for 6 h at 3,000 rpm, and it was found that the PO2 in the oxygen-enriched group decreased from 109 mmHg to 82 mmHg, with a loss of 27 mmHg of the partial pressure of the gas, whereas the PCO2 decreased from 101 to 100 mmHg, with a loss of only 1 mmHg and only 1 mmHg was lost; in the normal group, PO2 decreased from 99 mmHg to 76 mmHg with a loss of 23 mmHg, while PCO2 decreased from 95 mmHg to 92 mmHg with a loss of only 3 mmHg. The results suggested that in the presence of large amounts of gas bubble precipitation, the type of gas whose content decreased in both groups of blood specimens was mainly oxygen, and carbon dioxide precipitation was very small (Figure 7).

**Figure 8:**
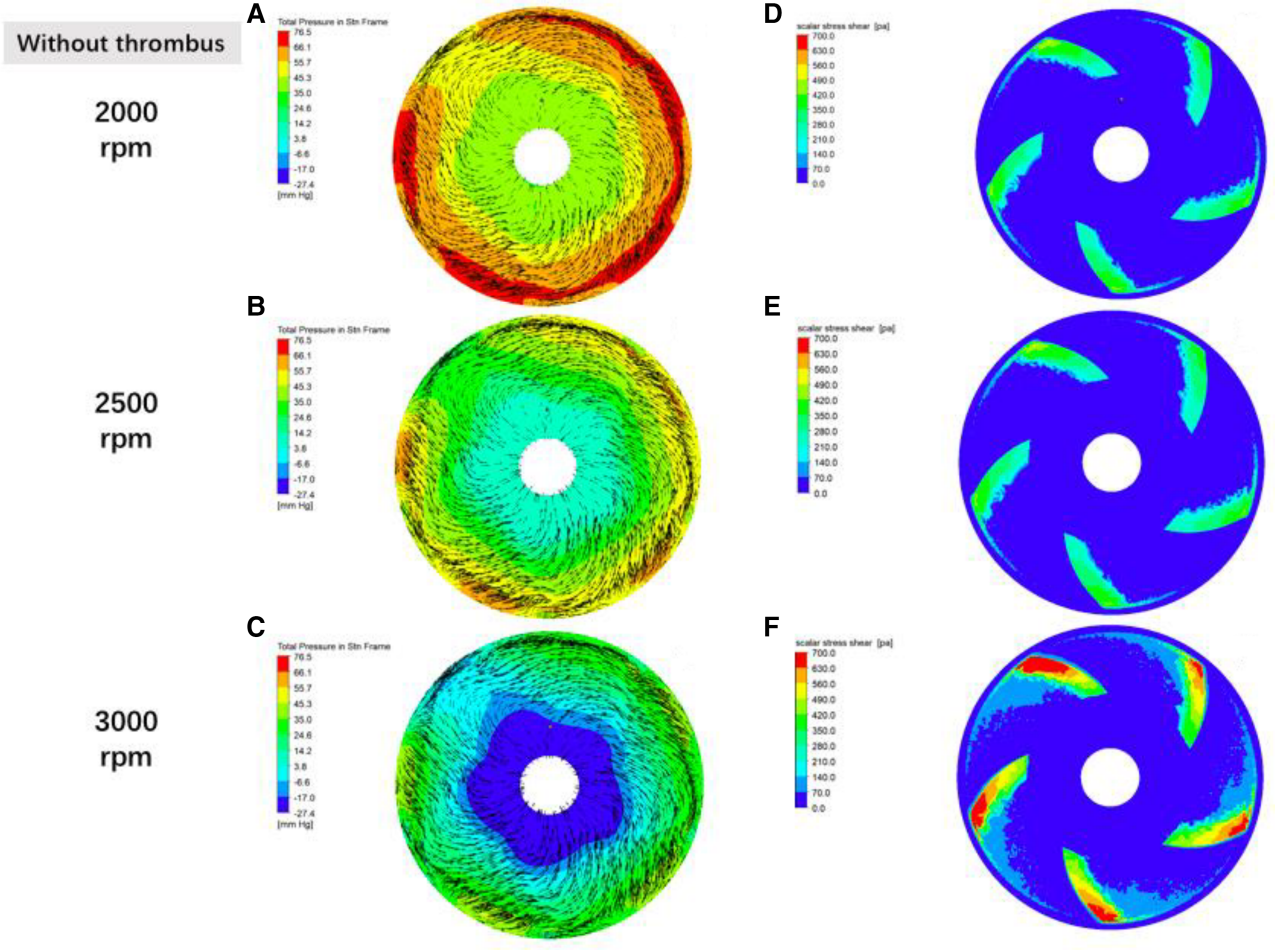
Distribution of fluid pressure, flow velocity vector and scalar shear between the rotor impeller and the lower worm shell of the blood pump in the absence of thrombus formation the gradient colors in the graphs of (**A**–**C**) are the fluid pressure, the black line segments are the velocity vectors, and the denser the black line segments suggests the higher flow rate; and the gradient colors in the graphs of (**D**–**F**) are the magnitude of the scalar shear force.

**Figure 9:**
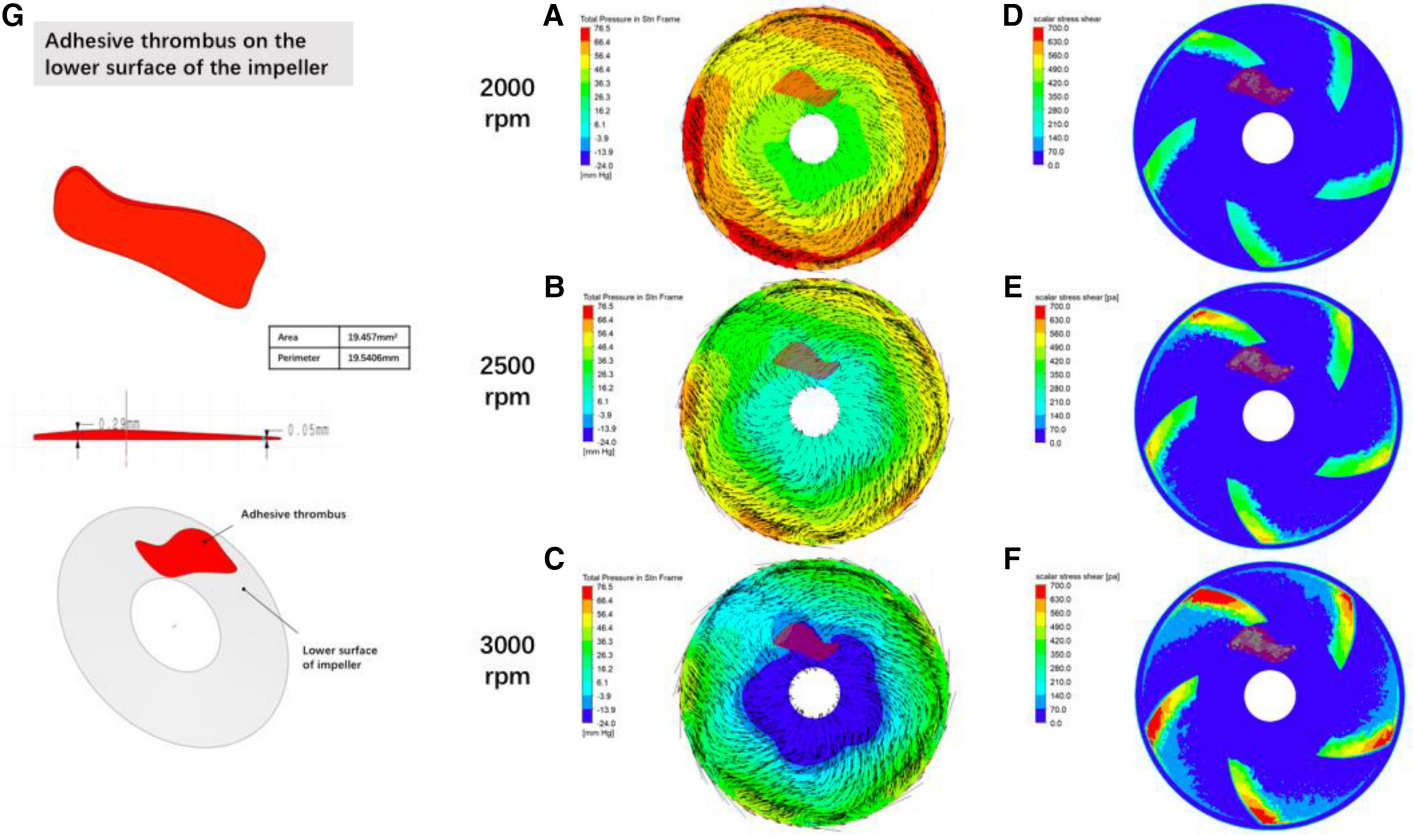
Distribution of fluid pressure, flow velocity vector and scalar shear between the rotor impeller and the lower worm case of the blood pump when thrombus forms on the lower surface of the rotor gradient colors in figures (**A**–**C**) are the pressure of the fluid, black lines are velocity vectors, and the denser the black line suggests a higher flow rate; gradient colors in figures (**D**–**F**) are the magnitude of the scalar shear force; figure (**G**) is the simulated thrombus on the simulation module.

Figure 8 demonstrates the effect of impeller speed on pressure, flow rate and shear in the secondary flow channel of a blood pump, and Figure 9 simulates the changes in pressure, flow rate and shear in the secondary flow channel during thrombus formation in the pump.

## 4 Discussion

In this study, the following results were obtained from the experiments on the *in vitro* gas production model: the high oxygen partial pressure group produced more air bubbles compared with the normal oxygen partial pressure group under the same rotational speed of the blood pump; the faster the rotational speed of the blood pump, the more air bubbles were produced under the same conditions of the oxygen partial pressure. The results of blood gas analysis before and after the operation of the blood pump showed that the partial pressure of oxygen in the blood decreased significantly, and the partial pressure of carbon dioxide did not decrease significantly. Computational fluidics studies of the prototype suggested that higher speeds produced lower rotor center pressures and higher scalar shear than lower speeds, and that the thrombosed group developed stronger flow field turbulence and local pressure changes than the non-thrombosed group.

A previous study assessed the postoperative intracardiac situation by ultrasound and found delayed intracardiac bubble production. The presence of a large number of gas bubbles in the arterial system is associated with a high risk of gas embolism as the bubbles travel throughout the body with the blood flow, which may lead to ischemia or even infarction of organs, such as cerebral infarction and myocardial infarction (14). Delayed gas bubble generation appeared after the signals of intra-pump thrombosis such as increased hemolysis and elevated blood pump power in experimental animals, suggesting that delayed gas bubble generation is an early warning of intra-pump thrombosis. Therefore, if delayed air bubble generation is found in the clinic, it should be highly valued and requires timely medical intervention. However, the extent to which thrombosis occurs is more likely to produce air bubbles needs further study.

In order to verify the hypothesis that an increase in pump speed has a facilitating effect on blood pump gas production, a fluid simulation study was carried out in this experiment, as shown in Figure 8, which shows the distributions of fluid pressure, flow velocity vector, and scalar shear between the lower surface of the rotor and the lower worm shell of the blood pump. It can be seen that with the increase of blood pump speed, the fluid pressure gradient in the secondary flow channel of the blood pump increases, which can promote the precipitation of gas from the blood, which coincides with the difference in the number of bubbles produced in the experimental group at 3,000 rpm and 2,500 rpm in Figure 5.

**Figure 10:**
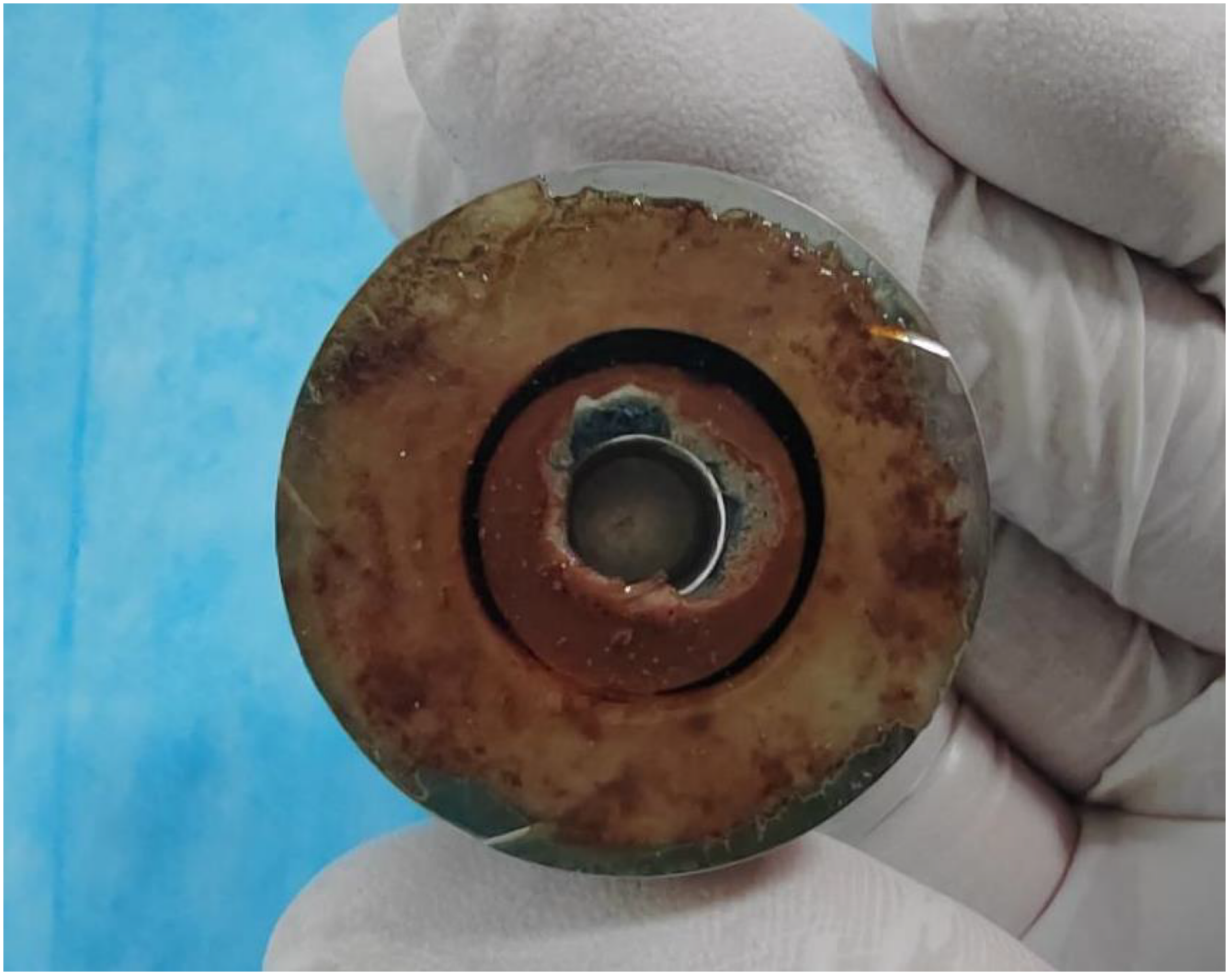
Thrombus formed on the lower surface of the magnetically levitated rotor.

The intracardiac gas bubbles in the experimental sheep did not occur at the early stage of LVAD implantation, suggesting that the smooth operation of the blood pump did not cause gas bubble precipitation. In this case, there was no murmur in the blood pump at the early stage of the experiment, and its power did not tend to increase, but with the aggravation of hemolysis, the power of the blood pump began to rise, the flow rate of the blood pump decreased, and a murmur appeared in the auscultation of the pump, which suggested that thrombus was formed in the pump, and at this stage, the free hemoglobin increased rapidly within a short period of time, until the controller appeared to have a low-flow alarm, a high power alarm, and the pump stopped, which is called the period of rapid progression. And it is coincidentally during this rapid progression period that body ultrasound detected a large number of air bubbles within the ventricles and artificial vessels. This phenomenon suggests that thrombosis accelerates hemolysis and promotes bubble production. To verify this conjecture, CFD was performed in this study with an attached thrombus model, as shown in Figure 9, which shows the fluid pressure, flow velocity vector, and scalar shear distributions between the rotor impeller and the lower worm shell of the blood pump when the thrombus is formed on the lower surface of the rotor. Comparing Figures 8, 9, it is easy to see that the formation of thrombus caused the flow field disturbance in the secondary flow channel, and the thrombus formed and adhered to the rotor surface of the blood pump resulting in a sudden change in the local pressure of the flow field, which may be an important driving factor for the gas production of the blood pump, and the pathological manifestations of the disassembled blood pump also confirmed the inference (Figure 10).

The fluid medium used in this study was fresh animal blood, which is the closest to human blood components, and was designed to simulate the conditions that drive bubble precipitation in a real blood environment. The blood samples were divided into a normal oxygen content group and an oxygen-enriched blood group according to the level of oxygen concentration inhaled by the animals at the time of blood sampling, and the experimental conditions were consistent except for the difference in partial pressure of blood oxygen. The results of the *in vitro* bubble experiments suggested that there was a significant difference in the gas production capacity of the two groups of blood, with the oxygen-enriched group producing significantly more bubbles than the normal group (Table 1, Figure 6), which also confirmed that blood oxygen content is an important factor affecting blood gas production, and that blood with a high partial pressure of blood oxygen is more likely to precipitate gas bubbles.

The partial pressure of oxygen in the arterial blood gas of sheep in this series of animal experiments was mostly between 100 and 110 mmHg, which belonged to the level of partial pressure of oxygen that was easy to produce gas from the results of the *in vitro* simulation, but it was not found that the gas production was a universal phenomenon, and we found that the gas-producing sheep had a common characteristic, which was the formation of intra-pump thrombus. Intra-pump thrombus may promote gas bubble precipitation in the following 2 ways: (1) The rotor is no longer smooth after thrombus formation, causing turbulence in the secondary flow channel and a sudden increase in the pressure gradient of the flow field; (2) The adherent thrombus increases the friction between the rotor and the bottom surface of the blood pump leading to frictional heat production and local temperature rise. Because there is substantial prior evidence that elevated temperature decreases the solubility of gases in solution, no control variables were investigated for temperature in this study.

Previous studies used a high-speed video camera to observe a transparent centrifugal blood pump model and investigated the effects of pump speed, fluid viscosity, and narrowing of the inflow channel on the generation of gas bubbles within the centrifugal pump, but the fluid medium applied was an aqueous glycerol solution, and the composition of the gases within the bubbles was not analyzed (15). This experiment investigated the effect of different oxygen levels on bubble precipitation in a real blood environment, which is an important addition to previous studies. The study on the type of gas precipitated also showed different results from previous studies, Biancucci et al.'s study (16) preferred the precipitated gas to be CO2, and indicated that significant changes in PO2 between 250 and 350 mmHg did not affect microbubble production, whereas in the present study, the oxygen-enriched group was distinguished from the normal group by using the animals to naturally inhale the oxygen, and the partial pressures of the oxygen in both groups were 109 mmHg and 99 mmHg, and even a pressure difference of 10 mmHg demonstrated significantly different gas-producing capabilities, so it is reasonable to suspect that Biancucci's non-blood-mediated study is not representative of real-world conditions.

Combined with the findings, there is reason to believe that the mechanism of gas formation is degassing rather than cavitation. Girod, in his *in vitro* experiments, applied a venturi in conjunction with different fluid pressures, and observed different types of bubbles and processes of bubble formation in the lumen distal to the stenosis. In the ultra-fast jet group, extremely tiny bubbles were observed to be ejected from the stenosis directly to the distal end, and existed for very short periods of time, whereas in the normal velocity jet group, no bubble formation was found at the stenosis, but rather larger diameter bubbles with longer retention times were found in the normal lumen at its distal end, the study is good evidence that the conditions for cavitation and degassing are different. In this experiment, the bubbles seen by ultrasound were measurable in diameter and were able to be tracked, which is only possible in large bubbles, and in microbubble clusters formed by cavitation it is not possible to measure the size of the bubbles or to track them by the ultrasound machine. In addition, from the CFD simulation results can also identify the two different gas production mechanisms, from Figure 8C, we can see that the blue zone in the center of the impeller is the center of the low-pressure, assuming that the end-diastolic pressure of the heart is close to 10 mmHg, then the scalar pressure in the blue zone can be reduced to −27.4 mmHg, which is far away from the scalar saturated vapor pressure of the water at 37 ℃ (−712.88 mmHg) and a large gap a large gap, so the mechanism of the gas production phenomenon simulated in this prototype is degassing rather than cavitation.

Although this study conducted a preliminary exploration of the gas production mechanism of the blood pump implantation model and gained some insights, there are still limitations. Firstly, the number of *in vitro* experiments is relatively small, and there may be a bias in the results, so it is necessary to increase the sample size in the next study to clarify the reproducibility of the study; secondly, the research model for the effect of thrombus formation in the pump on the production of gas is relatively single, and it is limited to the stage of the computerized flow simulation, and it should be integrated with the thrombus formation and the gas production mechanism of the blood pump implantation model in future studies. study, the thrombus model should be applied to the animal *in vivo* study, the results will be more convincing; finally, due to policy constraints, it was not possible to replicate the experimental results using HM3 (HeartMate 3 LVAD, Abbott), and it is planned to carry out the supplement of this experiment in the future when the conditions are ripe.

## 5 Conclusion

After preliminary explorations of *in vitro* gas production experiments and CFD in the blood environment of implanted LVADs, it was found that blood gas production was associated with higher partial pressures of blood oxygen, blood pump rotation speeds, and intrapump thrombosis, and that the mechanism of pump gas production was degassing of dissolved gases rather than cavitation of water, and that the type of gases released had a high likelihood of containing oxygen. Delayed gas production is an early warning factor for intrapump thrombosis.

## Data Availability

The original contributions presented in the study are included in the article/Supplementary Material, further inquiries can be directed to the corresponding authors.

## Supplementary material

Supplementary Video S1 A large number of air bubbles are seen in ventricular diastole pouring out of the blood pump inflow tube into the left ventricle.

Supplementary Video S2 A large number of air bubbles are seen gushing from the left ventricle into the ascending aorta during ventricular systole.
