# Supplementary material for: Discovery of delayed gas production after implantation of a continuous-flow left ventricular assist device and a preliminary exploration of the mechanisms of its occurrence
Source: Front Cardiovasc Med. 2024 Jul 23;11:1417005. doi: 10.3389/fcvm.2024.1417005 (PMC11300196; doi:10.3389/fcvm.2024.1417005)
Supplement: Supplementary file 1 [file Table1.pdf]

Dimensional parameters	Value
Outlet angle of backward curved impeller/°	75
Inflow tube inner diameter/mm	17
Rotor clearance from wall/um	200
Rotor height/mm	8
Outflow tube inner diameter/mm	8

Supplementary Table 1. Dimensional parameters of S prototype
